# Automated Placement of Scan and Pre-Scan Volumes for Breast MRI Using a Convolutional Neural Network

**DOI:** 10.3390/tomography9030079

**Published:** 2023-05-10

**Authors:** Timothy J. Allen, Leah C. Henze Bancroft, Kang Wang, Ping Ni Wang, Orhan Unal, Lloyd D. Estkowski, Ty A. Cashen, Ersin Bayram, Roberta M. Strigel, James H. Holmes

**Affiliations:** 1 Department of Medical Physics, University of Wisconsin-Madison, 1111 Highland Avenue, Madison, WI 53705, USA; 2 Department of Radiology, University of Wisconsin-Madison, 600 Highland Avenue, Madison, WI 53792, USA; 3 GE Healthcare, 3000 N Grandview Blvd, Waukesha, WI 53188, USA; 4 Carbone Cancer Center, University of Wisconsin-Madison, 600 Highland Avenue, Madison, WI 53792, USA; 5 Department of Radiology, University of Iowa, 169 Newton Road, Iowa City, IA 52242, USA; 6 Department of Biomedical Engineering, University of Iowa, 3100 Seamans Center, Iowa City, IA 52242, USA; 7 Holden Comprehensive Cancer Center, University of Iowa, 200 Hawkins Drive, Iowa City, IA 52242, USA

**Keywords:** breast MRI, deep learning, artificial intelligence, abbreviated breast MR, workflow, automation

## Abstract

Graphically prescribed patient-specific imaging volumes and local pre-scan volumes are routinely placed by MRI technologists to optimize image quality. However, manual placement of these volumes by MR technologists is time-consuming, tedious, and subject to intra- and inter-operator variability. Resolving these bottlenecks is critical with the rise in abbreviated breast MRI exams for screening purposes. This work proposes an automated approach for the placement of scan and pre-scan volumes for breast MRI. Anatomic 3-plane scout image series and associated scan volumes were retrospectively collected from 333 clinical breast exams acquired on 10 individual MRI scanners. Bilateral pre-scan volumes were also generated and reviewed in consensus by three MR physicists. A deep convolutional neural network was trained to predict both the scan and pre-scan volumes from the 3-plane scout images. The agreement between the network-predicted volumes and the clinical scan volumes or physicist-placed pre-scan volumes was evaluated using the intersection over union, the absolute distance between volume centers, and the difference in volume sizes. The scan volume model achieved a median 3D intersection over union of 0.69. The median error in scan volume location was 2.7 cm and the median size error was 2%. The median 3D intersection over union for the pre-scan placement was 0.68 with no significant difference in mean value between the left and right pre-scan volumes. The median error in the pre-scan volume location was 1.3 cm and the median size error was −2%. The average estimated uncertainty in positioning or volume size for both models ranged from 0.2 to 3.4 cm. Overall, this work demonstrates the feasibility of an automated approach for the placement of scan and pre-scan volumes based on a neural network model.

## 1. Introduction

MRI of the breast is an important clinical tool for evaluating the extent of disease in women with breast cancer, monitoring their treatment, and for cancer screening in high-risk women [1]. It is also used in the evaluation of breast implants. Additionally, cancer screening with abbreviated breast MR has seen rapid growth and requires highly optimized imaging protocols and workflow [2,3]. Currently, clinics need to choose between expedited workflow and robust image quality using highly tailored, patient-specific image prescription. In particular, image quality in breast MRI is well known to be highly variable. Clinical MRI vendors try to maximize image quality for each acquisition using scan-specific system settings that can include the x, y, and z linear shims, center frequency, transmit gain, and receive gain. Optimizing these settings on a scan-by-scan basis helps to maintain image quality by preventing several sources of potential artifacts. Consequences of poor system settings can include incomplete water excitation, flip angle inaccuracy, image distortion, or fat suppression failure. For example, recent work by Zhou et al. found a statistically significant relationship between the x, y, and z linear shims and the quality of fat suppression in breast MRI [4]. While their work focused on the linear shims, fat suppression quality relies on the totality of scan-specific system settings. Breast MRI involves complex anatomical geometry, a high number of air-tissue interfaces, and variable fat/water tissue composition that makes determining the scan-specific settings challenging [5].

Typically, scan-specific settings are determined using a short system calibration, also known as the pre-scan, at the start of each image series. The two general approaches to pre-scan are to either optimize scan settings globally across the entire scan volume or locally for a subregion within that volume. Since the axial breast image volume includes the breast, heart, lungs, arms, chest wall, and liver, the global approach results in optimized settings over this entire anatomy, which may be non-optimal over the breast tissue itself. Local pre-scan has been shown to be beneficial in breast MR by helping to achieve, for instance, a more homogenous B0 field using bilateral (two independent) pre-scan volumes, one for each breast [6,7].

Selection of the breast tissue for local pre-scan can be done using single or multiple pre-scan volumes. Manually aligning these volumes to the individual patient anatomy in all three dimensions is prone to error and intra- and inter-reader variability. The work by Zhou et al. also highlighted the variability in pre-scan placement by clinical technologists and how that variability could impact scan-specific system settings and image quality [4].

Scan volume selection is similarly prone to error and variability. The American College of Radiology (ACR) stipulates that the scan volume should be adequately positioned such that all of the breast tissue is covered including the axillary tail [8,9]. Variability in scan volume selection can also contribute to variable image quality by affecting signal-to-noise ratio, image resolution, and artifacts such as phase wrap. Thus, accuracy and consistency of scan volume placement are necessary for high-quality breast MRI.

An automated method for scan and pre-scan volume placement could limit volume placement variability and reduce the workload of MR technologists. Existing methods for scan volume placement have been investigated in a limited number of anatomies outside of the breast. Atlas- and template-based techniques have been shown for brain [10,11], knee [12], and liver [13]. Alternative approaches use segmentation algorithms or feature localization [14,15]. Ozhinsky et al. developed methods for placement of pre-scan volumes to help with fat suppression during spectroscopic imaging [15]. These existing techniques may be challenging to implement in breast MRI. Atlas-based approaches would require extensive anatomical atlases to capture the vast variation in breast anatomy. Other methods require robust segmentation or landmark identification, which is difficult in breast MRI due to the intermingling of fibroglandular and fat tissue and a lack of suitably consistent landmarks to guide volume placement.

More recently, machine learning approaches using deep, convolutional neural networks to automate volume prescription have been demonstrated [16,17,18,19,20]. However, these highly anatomically specific networks do not address the placement of either single or multiple pre-scan volumes. There is also a need for uncertainty estimates in deep learning predictions [21]. Techniques such as Monte Carlo dropout can provide uncertainty estimates but are not incorporated into the existing deep learning scan volume placement models [22,23,24,25,26].

Thus, there is a need for an automated, fast, and accurate technique for the placement of scan and pre-scan volumes in breast MRI. The purpose of this work is to demonstrate the feasibility of a breast-specific, deep-learning approach to achieve automated placement of these volumes.

## 2. Materials and Methods

### 2.1. Data Collection and Curation

**Figure 1:**
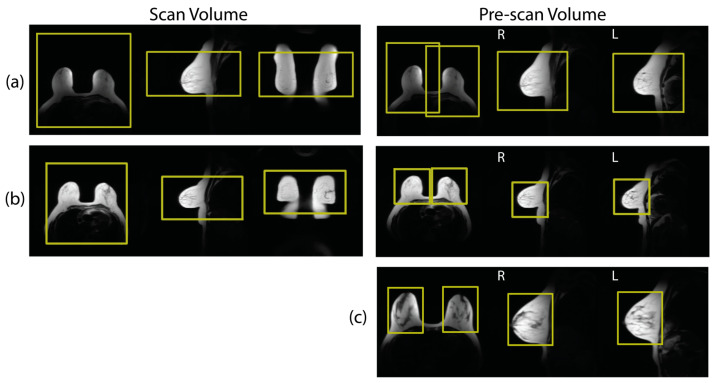
Examples (**a**,**b**) show scan volumes and pre-scan volumes as prescribed during clinical breast MRI. Both (**a**,**b**) come from the same patient but from exams on different days. Note that the placement of scan volumes was relatively consistent across both exams. However, the pre-scan volume placements were dramatically different between exams. Example (**c**) shows an example from a different patient with offline volumes generated by expert users that was used to train pre-scan volume placement.

Clinical breast MR exams (*n* = 413) from 12/07/2016 to 3/14/2018 were de-identified and retrospectively collected from 1.5 T and 3 T MRI scanners (*n* = 11) from a single MRI vendor (GE Healthcare, Waukesha, WI, USA) at a single healthcare system (UW Health, Madison, Wisconsin, USA). All exam collection was performed with institutional review board approval. For this feasibility work, patients with implants (*n* = 42) and substantial post-operative deformation due to surgery (e.g., mastectomy or lumpectomy, *n* = 21) were excluded (Table 1). Additionally, exams with incomplete imaging (*n* = 13), and insufficient scan volume placement (*n* = 4) were excluded leaving a final set of 333 cases. Patient age, indication for exam, and BI-RADS final assessment category was recorded. Each breast exam began with 3-plane anatomical scout imaging based on a 2D acquisition that collected images in axial, sagittal, and coronal orientations. Select scan parameters from the 3-plane anatomical scout are summarized in Table 2. If multiple scout image series existed in an exam, the most recent series was used in this work. Scout series were reviewed to ensure complete anatomical coverage and proper placement of breasts within the localizer images, as well as to identify any severe artifacts. Exams from scanner 6 in Table 2 did not achieve complete anatomical coverage and were excluded as part of the *n* = 13 cases with incomplete imaging. Examples of the typical image quality of scout images are shown in Figure 1.

The scan volumes and bilateral pre-scan volumes placed by the technologist during the clinical exams were also collected. A large amount of variation was observed in the clinically placed pre-scan volumes, which is consistent with the findings of Zhou et al. [4]. Figure 1a,b shows an example of repeated imaging with the same patient collected by chance in our exam data that demonstrates some of this variability. Due to this variability, the clinically placed pre-scan volumes were determined to be unsuitable to serve as training volumes for a machine learning network. An alternative set of bilateral pre-scan volumes were generated by a team of expert users in an offline configuration. The offline pre-scan volumes were placed by one medical physics graduate student (T.J.A.) and then reviewed in consensus by two experienced clinical physicists (J.H.H. and L.C.H.B). The desired volume characteristics were based on guidelines from the MR scanner vendor and included these specific goals: (1) Volumes should include the entire breast including the nipple. (2) The edges of the pre-scan volumes should be tight to the edges of the breast with around a 1 cm margin. (3) Inclusion of heart and lung tissues should be minimized. (4) There should be no overlap of the two bilateral volumes. An example placement following these criteria is shown in Figure 1c. The volumes were iteratively adjusted until all three reviewers agreed that the guidelines were met. It should be noted that the scan and pre-scan volumes come from two different sources: (1) scan volumes placed by technologists during the clinical breast MR, and (2) pre-scan volumes placed offline by expert users.

**Figure 2:**
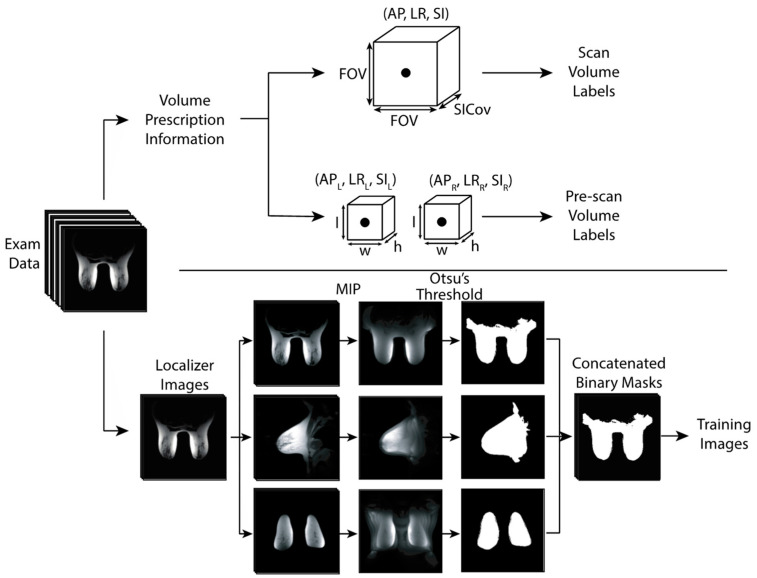
Parameterization of volume information (**top**) and image pre-processing of anatomical scout images (**bottom**) prior to neural network training. Note that the two shim volumes (represented by two cuboids) have a shared size. FOV: Axial field of view. SICov: Coverage in the SI direction. MIP: Maximum intensity projection.

Each volume (scan or pre-scan) was cuboid in shape as demonstrated in Figure 1 and Figure 2. The scan volume was square in the axial plane and was described by five placement parameters: the positions in the left-right (LR), anteroposterior (AP), superior/inferior (SI) directions, axial size (i.e., field-of-view, FOV), and extent of SI coverage. The bilateral pre-scan volumes were positioned independently, one over each breast, but the two volumes shared a common size. Therefore, the pre-scan volumes were parameterized by nine values: LR, AP, and SI position for each of the two volumes, and the size in each dimension.

### 2.2. Network Training

A convolutional neural network based on Alexnet [27] was trained for the placement of scan volumes and pre-scan volumes using the scout images as input. The network contains five convolutional layers and three fully connected layers. Dropout layers with a dropout rate of 0.5 were included between the fully connected layers. This dropout rate outperformed other tested dropout rates (0.25 and 0.75) and is consistent with the original Alexnet model [27]. For the purpose of this paper, the network in combination with a set of trained weights will be referred to as a placement model. The single network was trained under two conditions resulting in two placement models: (1) scan volume placement trained on clinical volumes and (2) pre-scan placement trained on the offline, consensus-based volumes. Although the network structure was identical for both training instances, the size of the final output layer was adjusted to output scan or pre-scan placement parameters (five vs. nine, respectively).

Before the scout images were used to train the neural network, several pre-processing steps were performed (Figure 2). The axial, sagittal, and coronal images were separated, and maximum intensity projections (MIPs) were calculated for each view. Each MIP was then binarized via Otsu’s method of automated thresholding to create three binary masks [28]. The three binary masks were then used as input into the network.

GPU-accelerated training was performed on an NVIDIA DGX A100 system with a batch size of 32. Using conventional approaches for training a deep learning network, weights are iteratively updated according to the loss function used. In this work, two loss functions were tested and the best-performing approach was used for the final model: (1) a 3D version of the generalized intersection over union (GIoU) [29] and (2) the root-mean-squared error (RMSE). The optimizer used was RMSprop [30]. Five times data augmentation was performed in the form of shifts in all three dimensions. Five-fold cross-validation of each placement model was performed. Using this technique, the training data (images and their associated volumes) are split into five groups with each group serving as the testing data in a round-robin fashion. This is thought to provide a more comprehensive assessment of the model performance across all available data compared to a simple training/validation/testing split.

Estimates of the model uncertainty were generated using Monte Carlo dropout. This technique generates multiple predictions for a given input using a random subset of the model weights for each individual prediction [25]. Monte Carlo dropout can increase model accuracy since distribution averages can be more accurate than any individual prediction. In this work, the mean of the 100 predictions was used as the final placement prediction and one standard deviation represented the prediction uncertainty.

### 2.3. Model Performance

Model performance was characterized by several metrics including the 3D intersection over union (IoU), 2D IoU, the absolute distance between volume positions in 3D space, and the percent difference in volume size. The 3D IoU quantifies the agreement of the known and predicted 3D volumes, while the 2D IoU quantifies the agreement between the 2D boxes obtained by projecting the 3D volumes into axial, sagittal, and coronal planes. For the bilateral pre-scan volumes, metrics are calculated for each volume independently. The dependence of model performance on the number of Monte Carlo predictions and across different scanners was evaluated. Finally, a comprehensive agreement score was developed by taking the sum of the IoU scores for the scan volume and the two pre-scan volumes. This metric is referred to as the combined IoU or cIoU and can range from 0 to 3 with higher scores indicating higher levels of agreement.

The time to prediction is important to characterize for any future clinical implementation. Therefore, the time to prediction starting from the raw, unprocessed images was measured. Timing measurements were obtained from 10 cases to get an estimate of the average time to prediction. The timing prediction study was performed on a 2018 MacBook Pro with a 2.6 GHz 6-Core Intel Core i7 processor. No GPU acceleration was used during the timing estimation experiments.

## 3. Results

### 3.1. Data Collection

A total of 333 exams were used for scan volume placement. The majority of these exams were performed to screen for breast cancer (72%), 16% were to evaluate the extent of known disease, 6% were for neoadjuvant chemotherapy response assessment, and the final 6% were in response to other imaging or clinical indications. Final BI-RADS assessment categories from 0 to 6 were included with at least five cases from each category. BI-RADS assessment categories of 1 and 2 comprised 71% of the exams. Patient age ranged from 20 to 77 years old with a mean age of 47. A total of 202 sets of pre-scan volumes were generated offline by the expert users and used to train the pre-scan model. Exam indication percentages, distribution of BI-RADS categories, and patient ages for the exams used for pre-scan volumes training were similar to those used for scan volume training.

### 3.2. Model Performance—Scan Volume

**Figure 3:**
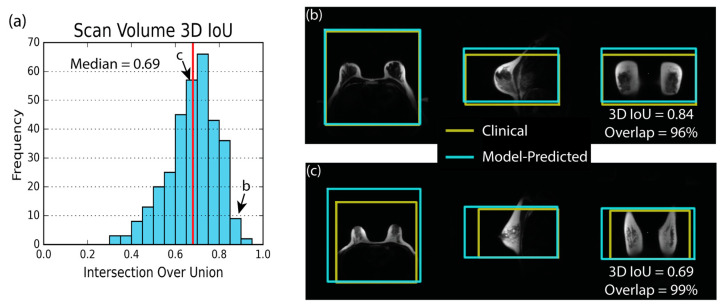
The scan volume 3D intersection over union (IoU) histogram, (**a**), and two placement examples, (**b**,**c**), demonstrate that the model can place a scan volume that is similar to scan volumes from the clinical exam. The red line indicates the median value of 3D IoU. In (**b**,**c**), the yellow box shows the technologist-placed volume generated during the clinical exam. The blue box shows the model-predicted volume. (**b**) shows an excellent case with a 3D IoU of 0.84 and 96% overlap of the clinically-placed volume. (**c**) shows the median example with a 3D IoU of 0.69 and an overlap of 99%.

Training with the RMSE loss function provided the highest median 3D IoU for scan volume placement. The histogram showing the 3D IoU distribution for all cases (Figure 3) demonstrates the overall good performance of the scan volume placement model with a median 3D IoU of 0.69. Figure 3 also shows examples of excellent and median scan volume placements using the RMSE-trained network and highlights the spatial relationship between the predicted volume and the anatomy. Distribution statistics for the various placement metrics can be found in Table 3. The median error in model predicted volume size was 2%, and the median absolute distance between the center of the model predicted volume and clinically placed volume was 2.7 cm. The median amount of overlap between the model-predicted and technologist-placed volumes was 84%. Results obtained using the GIoU loss function for training can be found in the Supplementary Materials (Table S1).

### 3.3. Model Performance—Pre-Scan

**Figure 4:**
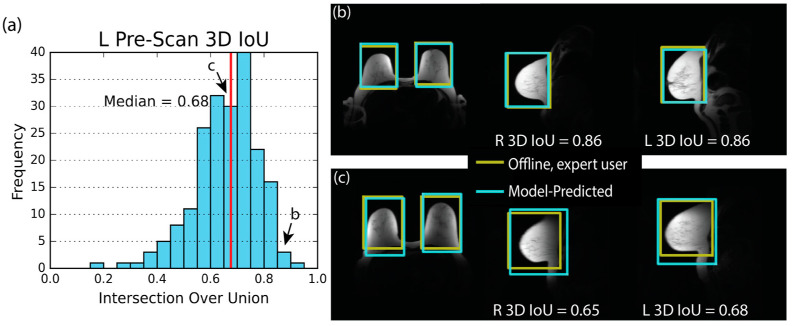
The pre-scan volume 3D intersection over union (IoU) histogram, (**a**), and the two examples, (**b**,**c**), demonstrate that the model can predict a pair of pre-scan volumes that closely match the offline, expert-placed pre-scan volumes. The histogram shows results of the left (L) volume only. However, the right (R) volume’s distribution is similar. The red line indicates the median value of 3D IoU. In (**b**,**c**), the yellow box shows the expert-user-placed volume generated in an offline setting. The blue box shows the model-predicted volume. (**b**) shows an excellent case with a high 3D IoU for both pre-scan volumes. (**c**) shows the median performing case.

Training for pre-scan placement using the GIoU loss function resulted in a higher median 3D IoU than with the RMSE loss function. The median 3D IoU for the left volume was 0.68 and the histogram displaying 3D IoU for all cases is shown in Figure 4. Examples of excellent and median placement are also shown with anatomical context. There was no significant difference in mean 3D IoU between the right and left volumes (*p* = 0.68). The median absolute distance between expert-placed and model-predicted volume centers was 1.2 cm. No significant difference between left and right mean absolute distance was observed (*p* = 0.80). The median volume size error was −2%. Distribution summaries of these metrics and given in Table 4. Results from the RMSE-trained model can be found in Table S2 (supplementary materials).

### 3.4. Uncertainty Estimate

Table 5 demonstrates uncertainty estimates for the predicted placement parameters derived using Monte Carlo dropout. For scan volume placement, the mean positioning uncertainty was 2.2 cm for AP position, 0.8 cm for LR position, and 1.4 cm for SI position. The mean uncertainties in the size parameters were 1.3 cm for axial size and 1.7 cm for SI coverage. Overall, 95% of predictions for all five placement parameters had an estimated uncertainty of less than 2.7 cm. For context, the average scan volume size is 33 × 33 × 19 cm^3^.

The mean uncertainty estimates for the AP, LR, and SI position of both left- and right-side volumes were 0.5 cm, 0.3 cm, and 0.6 cm, respectively. The shared size parameters of the pre-scan volumes had mean uncertainties of 0.7 cm, 0.4 cm, and 0.8 cm in the AP, LR, and SI directions, respectively. Overall, 95% of predictions for the nine pre-scan volume placement parameters had uncertainties under 1.0 cm. For context, the average pre-scan volume size is 13 × 11 × 16 cm^3^.

### 3.5. Overall Model Performance

Model performance increased with the number of Monte Carlo dropout predictions. The average 3D IoU of scan volume model was 0.62 when a single prediction was used, 0.67 when 10 predictions were used, and 0.68 when 20 predictions were used. Further increase in the 3D IoU after 20 predictions was less than 0.01 (Figure S1). The average 3D IoU of pre-scan volumes exhibited similar behavior.

**Figure 5:**
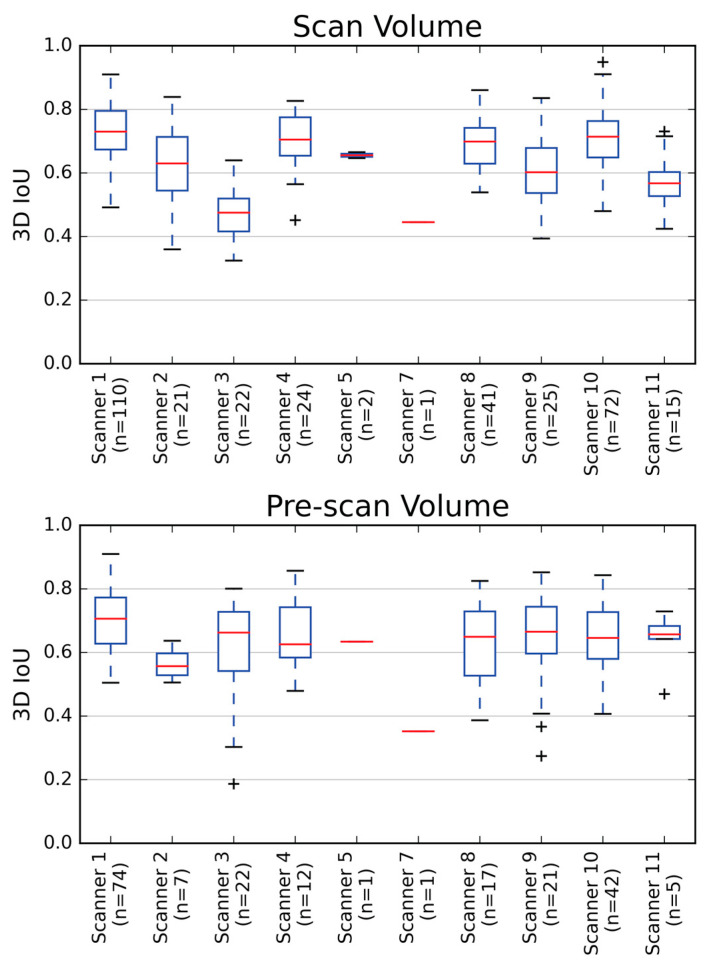
Average 3D intersection over unions (IoU) for both scan volume and pre-scan volumes were good across multiple scanners from which the data were obtained. 3D IoU performance on each scanner is displayed using box and whisker plots. Red horizontal lines represent the median values and ‘+’ indicates data outliers. The 3D IoU for the left pre-scan volume only is shown. The right pre-scan volume showed similar behavior. Generally, scanners with a larger number of cases tended to have higher 3D IoUs.

Figure 5 shows the scanner-by-scanner results for 3D IoU performance for both scan and pre-scan models. For scan volume placement on scanners with at least five cases, the average 3D IoU ranged from 0.73 on scanner 1 to 0.46 on scanner 3. The average 3D IoU for left pre-scan volumes on scanners with at least five cases ranged from 0.70 on scanner 1 to 0.65 on scanner 4. The behavior of the right pre-scan volumes was similar. Scanners with a higher number of cases tended to exhibit a higher 3D IoU.

The average and median cIoU across exams which had both scan and pre-scan volumes was 2.0. The 5th percentile of 3D cIoU values was 1.5 and the 95th percentile was 2.5. The standard deviation between the IoU of the three volumes was less than 0.1 for 98% of the cases. Figure S2 shows an exam with the median cIoU of our data.

Once trained, successive prediction of the scan and pre-scan volumes was achieved in an average of 4 s when only a single Monte Carlo dropout prediction was used. With 20 predictions, an average time of 16 s was required.

## 4. Discussion

In this work, we demonstrated the feasibility of using a convolutional neural network for the automated placement of scan and pre-scan volumes in breast MRI. Overall, we found good levels of agreement between the model-predicted and the human-placed volumes as evidenced by the values for the 3D IoU, the small difference in volume centers, and the small difference in volume size. This performance was achieved on data from multiple MR systems and with a variety of scout acquisition protocols. The uncertainty estimation feature provides a method for gauging model confidence, which was on the order of a few cm. Accurate placement predictions with estimated uncertainties were achieved using a reasonable number of Monto Carlo predictions and in a short amount of time. These promising results were obtained using a relatively small dataset, and additional training is likely to further improve model performance. Additional refinement of these models may allow for quick, reproducible, and automated placement of volumes needed for clinical breast MRI.

A novel feature of this work is the placement of multiple pre-scan volumes in addition to the scan volume. Values for the 3D IoU of the pre-scan volumes were generally slightly lower than for the scan volumes. However, IoU measurements are sensitive to the size of the volumes being assessed. Smaller volumes will produce smaller IoU values for a given error in position or size. The other placement metrics indicate that the pre-scan and scan volumes are similar in performance and the difference in IoU is likely due to the smaller size of the pre-scan volumes. The placement of bilateral pre-scan volumes such as those used in this work is expected to be more difficult than scan volumes due to the additional placement parameters (nine vs. five) necessary to predict pre-scan size and location. Therefore, the similar performance to scan placement seen in this work is encouraging.

Scan volume cases with the worst performance were observed to have anatomic variations that fell on the more extreme limits of our dataset, such as in cases with very large or small breast size. For the pre-scan volume, the worst cases were identified as subjects with small breast size and who were imaged with their arms above their head. In this setup, the breast tissue was stretched in the SI direction, leading to pre-scan volumes that were relatively small in the axial plane when compared to their length in the SI direction. With this shape, any location error in the axial plane led to a severe decrease in the 3D IoU metric. In general, for both the scan and pre-scan volumes, the error in location influenced the 3D IoU more than the error in size. While the volume size errors seemed to be quite high in some cases (from −40% up to 50%), it is useful to note that the volume scales quickly with small size errors. For example, a 1 cm overestimation of each side of a 10 × 10 × 10 cube leads to a size error of 33% and a 3D IoU of 0.75. Conversely, a 1 cm positional error in each direction for the same cube leads to an absolute distance error of only 1.7 cm, and a 3D IoU of 0.57. This example demonstrates the general fact that 3D IoU is more sensitive to distance error than size error. In accordance with this, the location errors in this work were also the dominant driver of low 3D IoU.

Another innovation of this work is the inclusion of uncertainty estimates in the placement predictions. Often, machine learning results do not include any measure of uncertainty, making it difficult to interpret the output. The models developed in this work can give an uncertainty estimate with every prediction. The estimates are academically useful in that they provide a measure of the overall model uncertainty. However, they could also be useful in a clinical imaging environment to alert the MR technologist to high levels of uncertainty and the need for further review or human intervention.

The models introduced here directly predict a full 3D volume. Previously described models required further processing to obtain the final scanning volume. For example, the approach described by Blansit et al. first generated landmark heatmaps and then predicted slice planes based on these heatmaps [19]. Alternatively, Geng et al. predicted 2D boxes on individual scout image slices and the final volume was that which contains all the predicted 2D boxes [27]. These additional processing steps introduce additional sources of error. For example, generating a 3D volume from 2D boxes will tend to overestimate the size of the 3D volume since it can only be as small as the largest 2D box. Because our approach predicts the 3D volume directly, these additional error sources are avoided. This is particularly salient for pre-scan volume placement since overestimating the size of pre-scan volumes may result in sub-optimal scan-specific system settings and poor image quality.

Further development of neural network-based volume placement could benefit clinical breast imaging. Observations of clinical technologists at our institution indicate that the entire volume placement process typically takes around 1–2 min but can take up to 3 min. This amount of time is substantial, especially when considered in the context of abbreviated breast MRI protocols that attempt to screen for breast cancer using shortened protocols with goals of reduced table time and ultimately decreased cost [2,3]. Conversely, the models introduced here can place a complete set of volumes in less than 20 s. Additionally, the models are expected to provide more reproducible volume placements than human technologists since the same input images will always yield the same result. Specifically, there should be no inter- or intra-operator variability with a neural network-based model. This could help achieve more consistent quality in breast MRI as well as more repeatable and reproducible quantitative MRI. However, the models are still sensitive to factors such as noise and patient position and, thus, the true placement consistency would need to be evaluated. Another potential use for the networks could be to assist in the training of MR technologists by comparing their own scan and pre-scan volumes with those generated by the placement models.

In this work, training the placement model using the RMSE loss function provided superior performance for the placement of scan volumes in terms of 3D IoU. This is inconsistent with the expected dependency where the use of a loss function similar to the performance metric would be expected to increase model performance. In the setting of 2D object detection, other researchers have commented on the instability of GIoU loss and the difficulty in obtaining accurate regression using GIoU, with some groups proposing more sophisticated loss functions to improve the GIoU loss [31,32].

This study has its limitations. First, the retrospective dataset was relatively small for neural network training, and cases with saline or silicone implants, as well as major surgical changes such as mastectomy, were excluded. While these are important clinical cases, they were not included in this initial feasibility work. Further research is necessary to assess the performance of the model when using a larger training dataset and in the setting of implants or mastectomy cases. This study only utilized single-shot fast spin echo-based scout acquisitions on a single vendor platform and future work is needed to expand the training data to include a broader range of imaging protocols and vendor implementations. Ultimately, the goal of the pre-scan volumes is to improve image quality by local optimization of pre-scan settings. This retrospective work focused on replicating human-placed volumes and did not look at how the models’ placements impacted image quality. Additionally, the retrospective data collection did not target specific patients who received repeat imaging. While a few patients with repeated imaging were collected by chance, there was not enough to fully analyze the variability in pre-scan placement by clinical technologists. This work is therefore unable to compare the variability in model-based pre-scan placements to the variability of clinical technologists. Further study of these models should include the acquisition of prospective data with an evaluation of the image quality and placement variability both using technologist- and network-derived pre-scan volumes. Finally, only one neural network, AlexNet, was used for testing. The study of additional models may lead to further improvement in volume prediction.

## 5. Conclusions

In summary, this work demonstrates the feasibility of a deep, neural network to accurately replicate human placement of scan and pre-scan volumes in breast MRI. Overall, the resultant network-based placements closely agreed with human users despite the use of a relatively small training dataset. Further improvements are anticipated with the inclusion of additional training data. These models show promise for quick and accurate placement of pre-scan volumes, which may help achieve consistent image quality in breast MRI.

## Figures and Tables

**Table 1:** Collected Data and Excluded Cases.

Cases Collected	413
Exclusions	80
Implants	42
Surgical Changes *	21
Incomplete Data	13
Poor Scan Volume Placement	4
Inclusions	333

* Surgical changes refer to exams excluded from training due to morphological changes brought about by surgery (such as mastectomy or lumpectomy).

**Table 2:** Summary of curated breast MR exam data.

ID	Model	Field Strength (T)	Slice Quantity	FOV (cm)	ScanVolume	Pre-Scan Volume
1	SIGNA HDxt	1.5	30–44	40	110	74
2	SIGNA Artist	1.5	45	38–46	21	7
3	Optima MR450w	1.5	35–60	40	22	22
4	Optima MR450w	1.5	45	38–44	24	12
5	SIGNA HDxt	1.5	45	40	2	1
6	Discovery MR750	3	37	44–46	0	0
7	SIGNA Premier	3	45	44	1	1
8	SIGNA Architect	3	45	44	41	17
9	SIGNA PET/MR	3	81	44	25	21
10	Discovery MR750w	3	40–44	40	72	42
11	SIGNA Premier	3	45	44	15	5
Total					333	202

FOV: Axial field of view. All exams from scanner 6 were excluded due to incomplete anatomic coverage of the scout images.

**Table 3:** Scan Volume Placement Metrics.

Metric	5th %	Median	95th %
3D IoU	0.46	0.69	0.85
Axial IoU	0.61	0.81	0.95
Sagittal IoU	0.53	0.73	0.89
Coronal IoU	0.6	0.78	0.92
Distance (cm)	0.9	2.7	6.6
Volume Error (%)	−30	2	45
Overlap (%)	57	84	99
RMSE (cm)	0.9	1.9	3.6

Results from scan volume model trained with RMSE loss function. IoU: intersection over union, Distance: absolute distance between the model-predicted volume centers and the technologist-prescribed volume center. RMSE: Root-mean-squared error between all 5 scan volume placement parameters. 5th % and 95th % stand for the 5th and 95th percentiles of the distributions, respectively.

**Table 4:** Pre-scan Volume Placement Metrics.

Parameter	Side	5th %	Median	95th %
3D IoU	R	0.45	0.65	0.83
	L	0.43	0.68	0.83
Axial IoU	R	0.52	0.75	0.90
	L	0.60	0.78	0.90
Sagittal IoU	R	0.51	0.73	0.87
	L	0.53	0.73	0.87
Coronal IoU	R	0.49	0.73	0.89
	L	0.55	0.75	0.90
Distance (cm)	R	0.5	1.3	2.9
	L	0.5	1.2	3.0
Volume Error (%)	N/A	−35	−2	56
RMSE (cm)	N/A	0.6	1.2	2.2

Results are from the scan volume model trained with generalized IoU loss function. IoU: intersection over union, Distance: absolute distance between the model-predicted volume centers and the expert-prescribed pre-scan volume centers. Side: Pre-scan volume placed on the right (R) or left (L) breast. RMSE: Root-mean-squared error between all nine pre-scan placement parameters. The column labels 5th % and 95th % stand for the 5th and 95th percentiles of the distributions, respectively.

**Table 5:** Uncertainty Estimates.

Scan Volume				Pre-Scan Volume			
Parameter	5th %	Mean	95th %	Parameter	5th %	Mean	95th %
AP Position	1.3	2.2	3.4	AP Position L	0.3	0.5	0.9
LR Position	0.5	0.8	1.3	LR Position L	0.2	0.3	0.6
SI Position	0.9	1.4	2.2	SI Position L	0.3	0.6	1.1
Axial Size (FOV)	0.8	1.3	2.1	AP Position R	0.3	0.5	0.8
SI Coverage	1.0	1.7	2.6	LR Position R	0.2	0.3	0.6
				SI Position R	0.3	0.6	1.0
				AP Size	0.5	0.7	1.1
				LR Size	0.3	0.4	0.6
				SI Size	0.5	0.8	1.1

All values are in cm. AP: Anterior-Posterior, LR: Left-Right, SI: Superior-Inferior, L: Left, R: Right.

## Data Availability

Data (localizers, scan volumes, and pre-scan volumes) used in this work are available on request at URL: https://radiology.wisc.edu/research/data/ (accessed on 8 May 2023). The signing of a data use agreement will be needed.

## Supplementary Materials

The following supporting information can be downloaded at: https://www.mdpi.com/article/10.3390/tomography9030079/s1, Figure S1: (a) The 3D intersection over union (IoU) of the models increases with the number of Monte Carlo (MC) predictions/iterations. Performing at least 20 predictions achieves similar 3D IoU as 100 predictions. (b) The average root-mean-squared uncertainty of the models can be estimated using 20 predictions which closely matches the uncertainty estimation at 100 predictions. The dotted lines show the average RMS uncertainty estimation when pure noise images are input into the model providing an upper bound on the uncertainty; Figure S2: (a) A median value of 2.0 was measured for the histogram of the combined intersection over union (IoU). This metric is the sum of the individual 3D IoU measures for the scan volume, right pre-scan volume, and left pre-scan volume. Thus, the minimum possible value is 0 and the maximum possible value is 3.0. The majority of cases in this work fell between 1.5 and 2.5. (b,c) An example with the median combined IoU score of 2.0 is shown and demonstrates good agreement between DL-predicted and clinical or human-generated volumes for scan volume or pre-scan volumes respectively; Table S1: Scan Volume Placement Statistics for the GIoU-trained Model; Table S2: Pre-scan Volume Placement Statistics for the RMSE-trained Model.
