# Supplementary material for: Automated Placement of Scan and Pre-Scan Volumes for Breast MRI Using a Convolutional Neural Network
Source: Tomography. 2023 May 10;9(3):967–80. doi: 10.3390/tomography9030079 (PMC10204486; doi:10.3390/tomography9030079)
Supplement: Supplementary file 1 [file tomography-09-00079-s001.zip › tomography-2362459-supplementary.pdf]

Supplemental Material

Table S1: Scan Volume Placement Statistics for the GIoU-trained Model.

Metric	5th %	Median	95th %
3D IoU	0.43	0.65	0.82
Axial IoU	0.55	0.8	0.93
Sagittal IoU	0.46	0.65	0.85
Coronal IoU	0.52	0.73	0.9
Distance (cm)	1.1	3.2	7.2
Volume Error(%)	-28	13	76
Overlap (%)	60	84	100
RMSE (cm)	1.1	2.3	4.2

IoU: intersection over union, Distance: absolute distance between model predicted volume centers and the technologist prescribed volume center. RMSE: Root mean squared error between all 5 scan volume placement parameters. 5th and 95th percentiles of the distributions, respectively.

Table S2: Pre-scan Volume Placement Statistics for the RMSE-trained Model

Parameter	Side	5th %	Median	95th %
3D IoU	R	0.42	0.65	0.82
	L	0.39	0.65	0.82
Axial IoU	R	0.47	0.75	0.90
	L	0.57	0.78	0.92
Sagittal IoU	R	0.49	0.73	0.87
	L	0.52	0.73	0.89
Coronal IoU	R	0.51	0.73	0.88
	L	0.56	0.76	0.91
Distance (cm)	R	0.5	1.3	3.5
	L	0.5	1.3	3.3
Volume Error (%)	N/A	-29	6	69
RMSE (cm)	N/A	0.6	1.2	2.3

Results are from scan volume model trained with generalized IoU loss function. IoU: intersection over union, Distance: absolute distance between the model predicted volume centers and the expert prescribed pre-scan volume centers. Side: Pre-scan volume placed on right (R) or left (L) breast. RMSE: Root mean squared error between all 9 pre-scan placement parameters. 5th and 95th % stand for the 5th and 95th percentiles of the distributions, respectively.

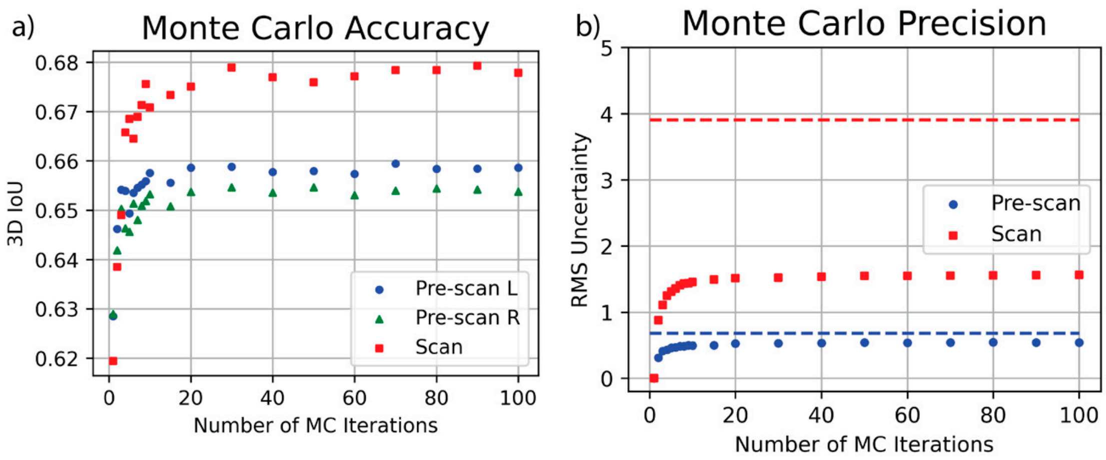

Figure S1: (a) The 3D intersection over union (IoU) of the models increases with the number of Monte Carlo (MC) predictions/iterations. Performing at least 20 predictions achieves similar 3D IoU as 100 predictions. **(b)** The average root-mean-squared uncertainty of the models can be estimated using 20 predictions which closely matches the uncertainty estimation at 100 predictions. The dotted lines show the average RMS uncertainty estimation when pure noise images are input into the model providing an upper bound on the uncertainty.

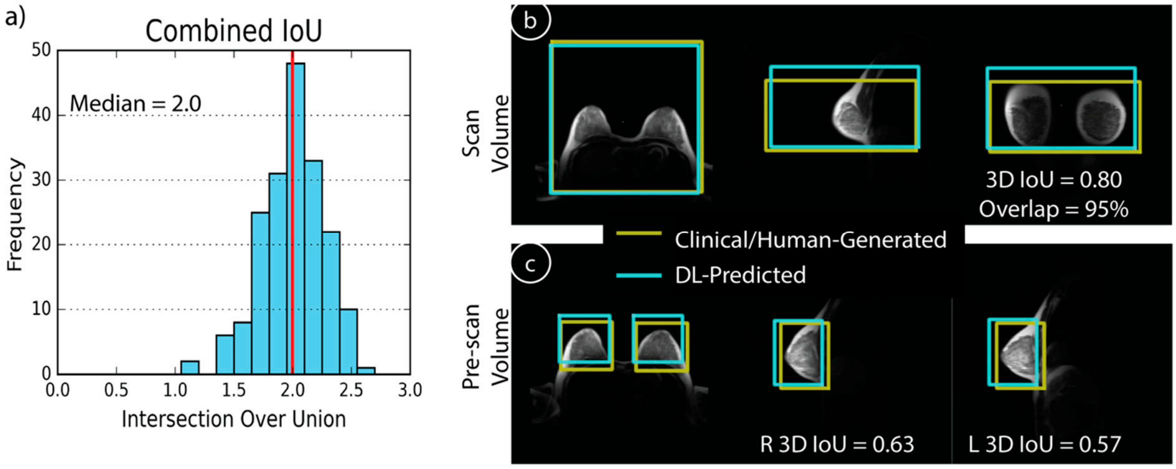

Figure S2: a) A median value of 2.0 was measured for the histogram of the combined intersection over union (IoU). This metric is the sum of the individual 3D IoU measures for the scan volume, right pre-scan volume, and left pre-scan volume. Thus, the minimum possible value is 0 and the maximum possible value is 3.0. The majority of cases in this work fell between 1.5 and 2.5. **b,c)** An example with the median combined IoU score of 2.0 is shown and demonstrates good agreement between DL-predicted and clinical or human generated volumes for scan volume or pre-scan volumes respectively.
